# Aromatic Compound-Dependent *Staphylococcus aureus* Is Safe in a Nasal Colonization Leukopenic Murine Model

**DOI:** 10.1155/2012/468539

**Published:** 2012-08-05

**Authors:** María S. Barbagelata, Lucía P. Alvarez, Cristian M. Dotto, Santiago M. Lattar, Daniel O. Sordelli, Fernanda R. Buzzola

**Affiliations:** ^1^Departamento de Microbiología, Parasitología e Inmunología, Facultad de Medicina, Universidad de Buenos Aires, C1121ABG Buenos Aires, Argentina; ^2^Instituto de Microbiología y Parasitología Médica, Facultad de Medicina, Universidad de Buenos Aires-Consejo Nacional de Investigaciones Científicas y Tecnológicas (IMPaM, UBA-CONICET), Paraguay 2155, Piso 12, C1121ABG Buenos Aires, Argentina

## Abstract

*Staphylococcus aureus* nasal carriage is a risk factor for individuals suffering from trauma, surgical procedures, invasive devices, and/or decreased immunity. Recently, we demonstrated that artificial nasal colonization with an attenuated *S. aureus* mutant reduced by bacterial interference with the colonization of pathogenic strains of *S. aureus*. This could be an optional tool to diminish the rate of *S. aureus* infections in hospitalized patients. The aim of this study was to construct a safe Δ*aroA* mutant of *S. aureus* and to discriminate it from nasal colonizing and osteomyelitis *S. aureus* isolates by *Sma*I pulsed-field gel electrophoresis (PFGE) typing. The Δ*aroA* mutant, named RD17, exhibited an LD_50_ (3.2 × 10^6^ colony-forming unit (CFU)) significantly higher than that of the parental strain (2.2 × 10^3^ CFU). The colony number of the RD17 mutants recovered from nares of leukopenic mice was similar to that observed in the animals of the control group. Therefore, the Δ*aroA* mutant was demonstrated to be safe due to maintaining low growth levels in the nares regardless of immune status of the animals. PFGE typing allowed the unequivocal identification of the *S. aureus* and differentiation of *aroA* mutants in nasal colonizing and osteomyelitis isolates. This information could be important to discriminate endogenous infections from laboratory strains of *S. aureus*.

## 1. Introduction

*Staphylococcus aureus* is part of the human microbiota and remains one of the most important community and nosocomial-acquired pathogens, with high rates of hospital-associated infections [1]. Although multiple body sites can be colonized, the anterior nares of the nose is the most common site of colonization [2]. Its prevalence in a healthy human population is around 30% [3]. Carriers of methicillin-resistant *S. aureus* (MRSA) have a higher risk of infection than those colonized by methicillin-sensitive (MSSA) strains [4, 5]. It has been demonstrated that most MRSA infections following initial colonization or infection are caused by identical strains [6].

Bloodstream infections are an important cause of morbidity and mortality during immunosuppressive conditions (diabetes mellitus, liver diseases, renal failure, corticotherapy, haemodialysis treatment, etc.), particularly for *S. aureus* nasal carriers [7, 8]. This susceptibility appears to be directly related to the severity and length of leukopenia [9]. Leukocytes, mainly neutrophils, are the main source of proinflammatory mediators and are essential for resistance to bacterial infections [10, 11].

Using a murine model of nasal colonization we demonstrated, recently, that an auxotrophic *S. aureus* mutant (named NK41) was able to interfere with the colonization of MRSA isolates belonging to Cordobes and Pediatric clones [12]. The auxotrophy of *S. aureus* NK41 mutant was achieved by insertion of a kanamycin resistance (Ka^*R*^) gene into the *aroA* gene, which encodes the enzyme 5-enolpyruvylshikimate 3-phosphate synthase (EPSPS). The EPSPS is part of the metabolic pathway of aromatic amino acids; therefore, bacteria with mutations in the initial steps of this metabolic pathway are auxotrophic to aromatic amino acids [13]. Herein, we constructed a Δ*aroA* mutant of *S. aureus,* and its safety was determined by nasal colonization of leukopenic mice. We also differentiated the Δ*aroA* (RD17) and *aroA*::Ka^*R*^ (NK41) mutants from nasal colonizing and osteomyelitis *S. aureus* isolates by pulsed field gel electrophoresis (PFGE) pattern. This additional knowledge is important to develop alternative strategies for the prevention of staphylococcal diseases.

## 2. Material and Methods

### 2.1. Bacterial Strain and Growth Conditions

Bacterial reference strains and plasmids and *S. aureus* attenuated mutants used in the present study are described in Table 1. Sixty-two single *S. aureus* isolates were obtained from 258 nasal swabs as a standard prophylactic procedure in patients at the time of admission to two hospitals of Buenos Aires City during the period 2009-2010. Also individual *S*. *aureus *isolates were obtained from 97 patients with osteomyelitis from seven hospitals in Argentina (four in Buenos Aires City, two in Buenos Aires Province, and one in the City of Santa Fe). Isolation and identification of *S. aureus* were performed according to routine culture procedures used in the Clinical Bacteriology laboratory [14]. Subcultures of single colonies of homogeneous size and pigmentation from primary isolation on blood agar plates (Britania, Buenos Aires, Argentina) were frozen in brain heart infusion (BHI) (Britania) broth with 20% glycerol (Promega, Madison, USA) at −20°C until further use. Species identification was confirmed by polymerase chain reaction (PCR) amplification of *S. aureus*-specific sequences according to Martineau et al. [15]. *S. aureus* isolates were tested for susceptibility to methicillin (oxacillin 1 *μ*g) (Britania) using the agar diffusion method according to the Clinical and Laboratory Standards Institute (CLSI) recommendations [16] and confirmed by PCR of *mec*A gene as performed by Fey et al. [17]. *Escherichia coli* strains were grown in Luria-Bertani (LB) medium (Britania) supplemented with ampicillin (Amp) (50 or 100 *μ*g/mL) (Sigma Chemical Co., St. Louis, USA), isopropyl-*β*-D-thiogalactopyranoside (IPTG) (0.5 mM) (Promega), and 5-bromo-4-chloro-3-indolyl-*β*-D-galactopyranoside (X-Gal) (20 mg/mL) (Promega) as needed for maintenance of plasmids. For selection of chromosomal markers or maintenance of plasmids, *S. aureus *antibiotic concentrations were the following: chloramphenicol (Cm) 10 *μ*g/mL (Sigma Chemical Co.), erythromycin (Em) 5 *μ*g/mL (Sigma Chemical Co.), and X-Gal, 40 mg/mL. For phenotype characterization assays, colonies were replicated onto defined minimum medium (DMM) agar plates for *S. aureus* as described by Pattee and Neveln [18]. Briefly, DMM agar plates were supplemented with the aromatic amino acids tryptophan (Trp) (0.05 mM), phenylalanine (Phe) (0.24 mM), and tyrosine (Tyr) (0.28 mM), as well as the precursors *p*-aminobenzoic acid (PABA) (0.05 mg/L) and 2,3-dihydroxybenzoic acid (DHB) (10 mg/L). All reactives utilized to prepare the DMM agar plates were purchased from Sigma Chemical Co. An *aro *mutant proliferates only in minimal medium supplemented with the three aromatic amino acids and PABA and DHB.

### 2.2. Generation of Deletion aroA Mutant

To obtain *S. aureus* Δ*aroA* mutant, four primers were designed (Table 2) that amplified two fragments of 625 and 980 bp that flanked the sequences of the *aroA* gene to the left (primers Aro-A and Aro-B) and the right (primers Aro-C and Aro-D), respectively. Primer Aro-C has a 16-base complementary region with primer Aro-B to allow the products of the first PCR anneal. A second PCR was performed with primers Aro-A and Aro-D to obtain a single fragment using the first PCR products as template. Then, 1 *μ*L of each of the first PCR products was mixed with 10 pmol of the outside primers and amplified by PCR. The fusion product (~1.6 kb) was purified and cloned in the pGEM-T Easy Vector (Promega). The plasmid was digested with *Bam*HI and *Sal*I (Promega) to purify the cloned fragment, and, finally, the fragment was fused by ligation into the shuttle plasmid pMAD (to yield pMAD-DEL). Blue and Amp^R^ colonies were selected on LB plates complemented with Amp and X-Gal. Plasmids were obtained from the selected transformants to verify by PCR the loss of the 650 bp fragment from the *aroA* gene yielding a 1.6 kb deletion fragment. This construction was named pMAD-DEL. The resulting plasmid was electropored into RN4220 *S. aureus *to generate transformants. Electrocompetent RN6390 was subsequently transformed with pMAD-DEL isolated from RN4220. pMAD contains a temperature-sensitive origin of replication and an Em resistance gene in gram-positive strains [19]. Homologous recombination experiments were performed as previously described [20]. RN6390 *S. aureus* strains with the desired fragment of PCR product were replicated onto DMM agar plates [18] supplemented or not with Trp, Phe, Tyr, PABA, and DHB to check the aromatic amino acid auxotrophic phenotype.

### 2.3. Complementation

A 1.4 kb fragment encompassing the *aroA* gene from *S. aureus *RN6390 was amplified by PCR using primers Fw-aro*Comp* and Rv-aro*Comp *(Table 2). The PCR fragment was restricted and ligated into vector pALC1743 (kindly provided by A. L. Cheung) after deletion of the *gfp* gene and then transformed into *E. coli *DH5*α* (Invitrogen, Carlsbad, CA) [21]. Restriction analysis and DNA sequencing confirmed the orientation and authenticity of the cloned gene. The recombinant plasmid was electroporated into the Δ*aroA* RN6390 *S. aureus *mutant (RD17), and Cm-resistant colonies were selected. Transformants were tested for restoration of the wild-type phenotype.

### 2.4. Determination of the Bacterial Virulence in Mice

CF-1 outbred mice were bred and maintained in the vivarium of the Department of Microbiology, School of Medicine, University of Buenos Aires in accordance with the guidelines set forth by the US National Institutes of Health [22]. For 50% lethal dose (LD_50_) studies, 6-week-old male CF-1 mice were injected intraperitoneally with 0.5 mL of a suspension ranging from 10^1^ to 10^9^ CFU of bacterial strain and 2% (w/v) Brewer's yeast (Sigma Chemical Co.) in BHI broth [23]. Three groups, each comprising 10 mice, from three separate tests received serial log dilutions of each bacterial strain. The estimation of the LD_50_ was made after 7 days using a software for probit analysis (PASW 18, IBM Software, Inc.) [24].

### 2.5. Nasal Colonization in Leukopenic Murine Model

CF-1 female mice weighing 27 to 32 g were used for the experiments. Mice were rendered leukopenic by injecting cyclophosphamide (Sigma Chemical Co.) (200 mg/kg/day) on days 4 and 2 before challenge. Previous studies have shown that this regimen produces leukopenia in this model for 5 days [25]. At time 0, groups of 10–15 mice were challenged by the intranasal route with 10 *μ*L of suspension containing approximately 10^7^ CFU of the RD17 (∆*aroA* mutant) or Sa14 (MRSA isolate). To evaluate nares colonization, mice were CO_2_-euthanized 24 hours after bacterial challenge and cultures were made from their nasal tissues. The area around the nasal region was wiped with 70% ethanol, and the nose was excised and homogenized in 400 mL tryptic soy broth (TSB) (Britania) using a tissue grinder. The lungs also were excised and homogenized separately in 2 mL of sterile distilled water. Tenfold serial dilutions of the tissue homogenates and blood samples were plated onto tryptic soy agar (TSA) (Britania) plates. Animals not treated with cyclophosphamide, but challenged under the conditions as the leukopenic mice, were used for control.

### 2.6. Pulsed-Field Gel Electrophoresis (PFGE) Typing

The clonality of the *S. aureus* clinical and nasal isolates, the *S. aureus* reference strains, and their derived attenuated mutants was assessed by PFGE of *Sma*I-digested (Promega) chromosomal DNA fragments [27] using a CHEF-DR II apparatus (BioRad Laboratories, CA, USA) as previously described [28]. Reference strains representative of the prevalent MRSA clones in Buenos Aires (Cordobes, Pediatric, and Brazilian, resp.), were included [26]. The similarity between PFGE types was evaluated by the Dice coefficient. The resultant similarity matrix was analyzed by the unweighted pair group method using arithmetic averages (UPGMA), and data were analyzed with the TREECON software for Windows [29].

### 2.7. Statistical Analysis

In order to obtain a statistical assessment of virulence for mice of the *S. aureus* RN6390 and RD17 mutants, the 7-day survival ratios from three separate tests were pooled for estimation of the LD_50_ by a computrrized program for probit analysis (PASW 18, IBM Software, Inc.). Nonparametrical data was analyzed with the Mann-Whitney test using the GraphPad Prism version 4.00 software for Windows. *P* values lower than 0.05 were considered statistically significant.

## 3. Results

### 3.1. Characterization of RN6390 Δ*aroA* Mutant

After the analysis of 950 colonies, only 2 named RD17 and RD89 presented the loss of a 648 bp fragment between the 659 and 2201 sites of the *aroA *gene yielding a 1.6 kb PCR product. Then, in order to characterize the auxotrophic phenotype, the isolated mutants were replicated onto DMM agar plates for *S. aureus *[18] with or without aromatic amino acids Trp, Phe, Tyr and its precursors PABA and DHB. The bacteria that failed to grow after 20 hours of incubation on DMM plates without addition of the three aromatic amino acids and PABA and DHB were considered Aro-deficient. Therefore, the deletion of the *aroA* gene encoding the EPSPS led to an *aroA* auxotrophy of the RD17 and RD89 mutants. Because the RD17 and RD89 mutants exhibited the same phenotype and genotype, subsequent experiments were performed only on RD17. Complementation assay restored the wild-type phenotype of the RD17 mutant. The growth rates of the Δ*aroA* mutant and its parental strain RN6390 were similar in TSB medium (data not shown). In addition, RD17 mutant exhibited a very stable phenotype, since its reversion frequency performed as described previously was <1 × 10^−12^ [13].

To determine the attenuation of RD17 mutant in mice, the probit analysis comparing the LD_50_ with the Δ*aroA* mutant and its parental strain was performed. The LD_50_ of the parental RN6390 strain (2.2 × 10^3^ CFU ±95% CI: 2.6 × 10^1^–8.5 × 10^3^ CFU) was statistically significant (*P* < 0.0001) different from the LD_50_ of the RD17 mutant (3.2 × 10^6^ CFU ±95% CI: 1.6 × 10^6^−9.7 × 10^6^ CFU). This statistical increase in the LD_50_ of the RD17 mutant confirmed its attenuation in mice.

### 3.2. Nasal Colonization in Leukopenic Murine Model

**Figure 1:**
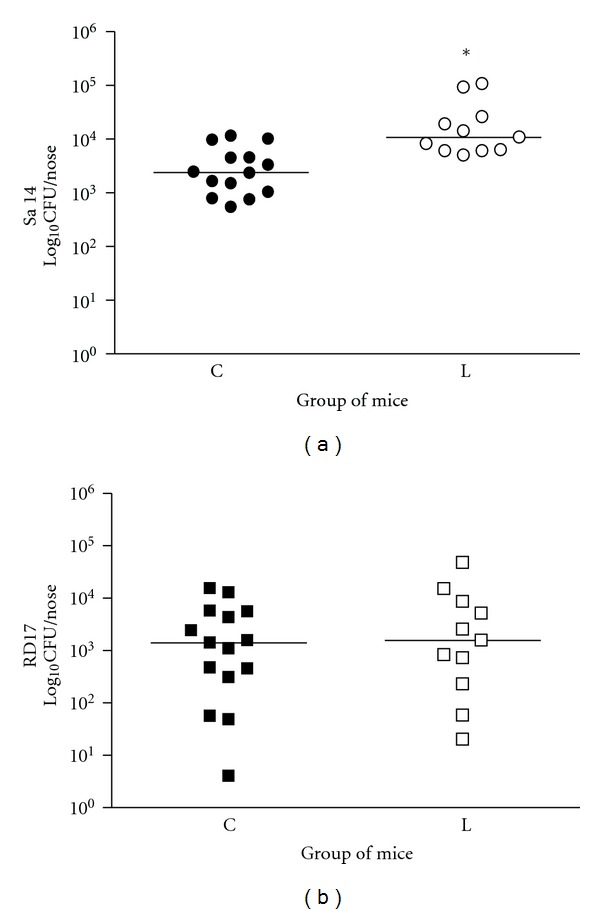
Nasal colonization in a mice leukopenic model. Groups of 10–15 leukopenic and control mice were inoculated intranasally with a suspension of 10^7^ CFU of the Sa14 (panel a) isolate or RD17 (panel b) attenuated mutant. Challenged mice were sacrificed and their nose tissue cultured to determine CFU. The horizontal lines represent the median values. Panel (a) the leukopenic (L) mice showed a significant increase in the CFU number of the Sa14 isolate (median = 10800 CFU/nose) compared with that of control group (C) (median = 2384 CFU/nose). *P < 0.001, Mann-Whitney test. Panel (b): animals inoculated with the RD17 mutant showed similar CFU number in the L group (median = 1552 CFU/nose) as well as in the C group of mice (median = 1400 CFU/nose). *P* > 0.05, Mann-Whitney test.

The safety of the Δ*aroA* mutant was evaluated using leukopenic mice model. To render the nasal colonization, the RD17 mutant and an epidemiologically unrelated MRSA nasal isolate (Sa14) were administrated into the nose of leukopenic groups of mice as we described previously [12]. CF-1 mice were treated with cyclophosphamide to render them leukopenic. Mice receiving cyclophosphamide had significantly lower white blood cells counts (331 ± 185 cells/mL) than mice receiving the saline control (3044 ± 501 cells/mL) (*P* < 0.0001). The number of Sa14 (MRSA) colonies recovered from the noses of leukopenic mice group was significant higher than that observed in the control group (Figure 1(a)); in contrast, the number of RD17 mutant colonies recovered was similar between the groups (Figure 1(b)). No bacteria was recovered from blood or lungs of neither the groups of mice (data not shown). Therefore, the RD17 mutant maintained low growth levels in the nose regardless of immune status of the animals.

### 3.3. Pulsed-Field Gel Electrophoresis (PFGE) Typing

**Figure 2:**
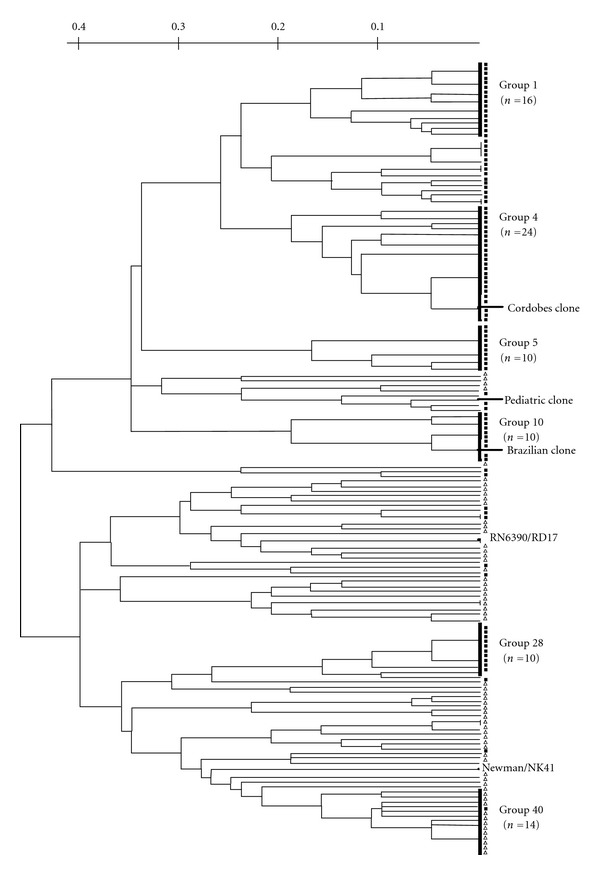
Dendrogram of *S. aureus* attenuated mutants (RD17 and NK41), their parental (RN6390 and Newman) strains, and *S. aureus* nasal colonizing (Δ) and osteomyelitis (■) (*n* = 159) isolates. *Sma*I PFGE typing discriminated 40 different pulsotypes. The major groups were named 1, 4, 5, 10, 28, and 40. *S. aureus* strains representative of Cordobes, Pediatric, and Brazilian clones are indicated [26].

All *S. aureus* osteomyelitis and nasal colonizing isolates (*n* = 159), the obtained mutants (RD17 and NK41), and their parental strains (RN6390 and Newman) were discriminated into 40 fingerprint groups by PFGE typing (Figure 2). A dendrogram that included all patterns was constructed on the basis of the levels of similarity, and a cut-off point of 80% was considered to define the groups. *Sma*I PFGE band analysis revealed the presence of six major pulsotypes (named 1, 4, 5, 10, 28, and 40) that included 86 isolates. The remainders were evenly distributed in 33 groups of one to five isolates and one group with 9 isolates. All mutants and their parental wild-type strains exhibited a markedly different macrorestriction genotype when compared with those of nasal colonizing and osteomyelitis isolates (Figure 2). The number of genetic differences of the RD17 and NK41 mutant genomes (RN6390 and Newman background, resp.), compared with those *S. aureus* isolates was three or more, which made them clonally different according to the criteria set by Tenover et al. [27]. These results show that RD17 and NK41 mutants could be discriminated from osteomyelitis and nasal colonizing *S. aureus* isolates using *Sma*I macrorestriction PFGE typing.

## 4. Discussion

The disruption of the *aroA* gene that encodes for EPSPS enzyme leads to an auxotrophy in *S. aureus* for DHB, PABA, and aromatic amino acids. The inhibition of EPSPS results in shikimate accumulation, inhibition of synthesis of aromatic amino acids and secondary metabolites causing cell death [30]. We constructed previously *S. aureus aroA* mutants by transposon mutagenesis with Tn*917* (FB306) and by insertion of the Ka^*R*^ gene into the *aroA* gene by allelic replacement mutagenesis (NK41). Both mutants were tested in two *in vivo* infection models with different purposes [12, 13]. Recently, we demonstrated that the NK41 mutant interfered with the nasal colonization of clinical isolates of *S. aureus* representative of prevalent clones in Argentina. It is important to note that the FB306 and NK41 mutants have inserted in their genome an antibiotic resistance gene allowing their easy selection and *in vitro* identification. However, this feature is a disadvantage for its potential use in vertebrate. For this reason we constructed a Δ*aroA *mutant of *S. aureus* RN6390 strain by allelic exchange. The obtained mutant (RD17) failed to show any measurable reversion and remained attenuated *in vivo*.

In the present study, the nasal colonization of leukopenic mice represented a risk state for disseminated infections by *S. aureus*. In this model we observed that the MRSA Sa14 isolate multiplied in noses more easily due to decreased host defenses and this increase was statistical significance with respect to the control group (*P* < 0.001). In contrast, leukopenic animals challenged with the attenuated mutant RD17 showed similar levels of nasal colonization as the control animals group; therefore, the RD17 mutant was safe under the conditions studied. Indeed, the RD17 mutant maintained the low growth levels in the nose regardless of the immune status of animals.

On the other hand, it was of interest to differentiate laboratory strains and their mutants (RD17 and NK41) from *S. aureus* isolated from patients with osteomyelitis and nasal carriers. This information will be required for future studies. We performed *Sma*I PFGE typing because it is still the most discriminative of the available genotypic methods for *S. aureus* [31]. The analysis indicated that the RD17 and NK41 mutants were unequivocally discriminated from nasal colonizing and osteomyelitis *S. aureus* isolates under investigation. Moreover, both *aroA* mutants showed pulsotypes totally different to those observed for *S. aureus* representative of Brazilian, Cordobes, and Pediatric clones. A major concern to use an attenuated live mutant *in vivo* is the possibility that new cases of the disease may be caused by the revertant strain. The deletion of *aroA* gene described in this paper showed the stability of the RD17 mutant in mice and its inability to cause disseminated infections in leukopenic nasal colonized mice. Therefore, the spreading of spontaneous derivative RD17 mutants would be an unlikely event to occur.

In the last years emergence of mupirocin resistance among MRSA and MSSA isolates has been reported [32–36]. This is a worrying situation in addition to the emergence of multiresistant *S. aureus *strains [37–39]. Based on our results we postulate that *aroA* mutant of *S. aureus* could be utilized as an alternative strategy to reduce the staphylococcal nasal carriage.

## Figures and Tables

**Table 1:** Bacterial strains and plasmids used in this study.

Strain or plasmid	Comment	Source
RN4220	Mutant strain of *Staph. aureus* 8325-4 that accepts foreign DNA	
RN6390	*ag* *r* ^+^ laboratory strain related to 8325-4, maintains haemolytic pattern when propagated on sheep
	erythrocytes
Newman	*S. aureus* polysaccharide capsular serotype 5 (CP5)	
NK41	Newman *aroA*::Ka^*R*^	
RD17; RD89	RN6390 Δ*aroA *	This study
DH5*α*	*E. coli * host cloning vectors	Invitrogen
pMAD	*E. coli*—*S.aureus* (*Listeria*) shuttle vector with the *bgaB* gene encoding a *β*-galactosidase. Amp^*R*^/Em^*R*^	
pMAD-DEL	pMAD plasmid containing the mutant allele for deletion of the *aroA* gene	This study
pGEM-T Easy	Amp^*R*^, *lacZ*′, f1 *ori*, MCS, Mob^−^, cloning T vector	Promega
pALC1743	pSK236 (*gfp*_*uvr*_with* agr* P3 promoter)	

**Table 2:** Primers used in this study.

Name	Sequence^a^
Fw-aro*Comp *	5^′^-CTCTCTAGAACATTACAACATGCATGTGAAC-3^′^
Rv-aro*Comp *	5^′^-ACGCGTCGACTGCGTCATCGTTGTCAGTAGT-3^′^
Aro-A	5^′^-CTCGGATCCACATTACAACATGCATGTGAAC-3^′^
Aro-B	5^′^-TAATGATGGTCGGTTCCTT-3^′^
Aro-C	5^′^-TTCTAAGGAACCGACCATCAGCGAGCCTGTCAAAATCAA-3^′^
Aro-D	5^′^-ACGCGTCGACCATCGCCGTGTTCTATTTCC-3^′^

^a^Enzyme restriction sites are underlined; TCTAGA: *Xba*I, GTCGAC: *Sal*I, GGATCC: *Bam*HI.

## References

[1] DeLeo FR, Chambers HF (2009). Reemergence of antibiotic-resistant *Staphylococcus aureus* in the genomics era. *The Journal of Clinical Investigation*.

[2] Lowy FD (1998). Medical progress: *Staphylococcus aureus* infections. *The New England Journal of Medicine*.

[3] van Belkum A, Melles DC, Nouwen J (2009). Co-evolutionary aspects of human colonisation and infection by *Staphylococcus aureus*. *Infection, Genetics and Evolution*.

[4] Carbon C (1999). Costs of treating infections caused by methicillin-resistant staphylococci and vancomycin-resistant enterococci. *Journal of Antimicrobial Chemotherapy*.

[5] Huang SS, Platt R (2003). Risk of methicillin-resistant *Staphylococcus aureus* infection after previous infection or colonization. *Clinical Infectious Diseases*.

[6] Huang SS, Diekema DJ, Warren DK (2008). Strain-relatedness of methicillin-resistant *Staphylococcus aureus* isolates recovered from patients with repeated infection. *Clinical Infectious Diseases*.

[7] Donowitz GR, Maki DG, Crnich CJ, Pappas PG, Rolston KV (2001). Infections in the neutropenic patient–new views of an old problem. *American Society of Hematology*.

[8] Fowler VG, Miro JM, Hoen B (2005). *Staphylococcus aureus* endocarditis: a consequence of medical progress. *JAMA*.

[9] Pizzo PA (1999). Fever in immunocompromised patients. *The New England Journal of Medicine*.

[10] O’Neill LAJ (2002). Toll-like receptor signal transduction and the tailoring of innate immunity: a role for Mal?. *Trends in Immunology*.

[11] Scapini P, Lapinet-Vera JA, Gasperini S, Calzetti F, Bazzoni F, Cassatella MA (2000). The neutrophil as a cellular source of chemokines. *Immunological Reviews*.

[12] Barbagelata MS, Alvarez L, Gordiola M (2011). Auxotrophic mutant of *Staphylococcus aureus* interferes with nasal colonization by the wild type. *Microbes and Infection*.

[13] Buzzola FR, Barbagelata MS, Caccuri RL, Sordelli DO (2006). Attenuation and persistence of and ability to induce protective immunity to a *Staphylococcus aureusaroA* mutant in mice. *Infection and Immunity*.

[14] Bannerman TL (2003). *Staphylococcus, Micrococcus, and Other Catalase-Positive Cocci That Grow Aerobically*.

[15] Martineau F, Picard FJ, Roy PH, Ouellette M, Bergeron MG (1998). Species-specific and ubiquitous-DNA-based assays for rapid identification of *Staphylococcus aureus*. *Journal of Clinical Microbiology*.

[16] (2006). *Performance Standards for Antimicrobial Disk Susceptibility Tests; Approved Standard*.

[17] Fey PD, Saïd-Salim B, Rupp ME (2003). Comparative molecular analysis of community- or hospital-acquired methicillin-resistant *Staphylococcus aureus*. *Antimicrobial Agents and Chemotherapy*.

[18] Pattee PA, Neveln DS (1975). Transformation analysis of three linkage groups in *Staphylococcus aureus*. *Journal of Bacteriology*.

[19] Arnaud M, Chastanet A, Débarbouillé M (2004). New vector for efficient allelic replacement in naturally nontransformable, low-GC-content, gram-positive bacteria. *Applied and Environmental Microbiology*.

[20] Valle J, Toledo-Arana A, Berasain C (2003). SarA and not *σ*B is essential for biofilm development by *Staphylococcus aureus*. *Molecular Microbiology*.

[21] Kahl BC, Goulian M, Van Wamel W (2000). *Staphylococcus aureus* RN6390 replicates and induces apoptosis in a pulmonary epithelial cell line. *Infection and Immunity*.

[22] National, Research, and Council (1996). *Book Guide for the Care and Use of Laboratory Animals (NIH guide, revised)*.

[23] Mei JM, Nourbakhsh F, Ford CW, Holden DW (1997). Identification of Staphylococcus auerus virulence genes in a murine model of bacteraemia using signature-tagged mutagenesis. *Molecular Microbiology*.

[24] Finney D (1971). *Probit Analysis*.

[25] Craig WA, Redington J, Ebert SC (1991). Pharmacodynamics of amikacin in vitro and in mouse thigh and lung infections. *Journal of Antimicrobial Chemotherapy*.

[26] Lattar SM, Tuchscherr LPN, Centrón D Molecular fingerprinting of *Staphylococcus aureus* isolated from patients with osteomyelitis in Argentina and clonal distribution of the *cap*5(8) genes and of other selected virulence genes.

[27] Tenover FC, Arbeit R, Archer G (1994). Comparison of traditional and molecular methods of typing isolates of *Staphylococcus aureus*. *Journal of Clinical Microbiology*.

[28] Quelle LS, Corso A, Galas M, Sordelli DO (2003). STAR gene restriction profile analysis in epidemiological typing of methicillin-resistant *Staphylococcus aureus*: description of the new method and comparison with other polymerase chain reaction (PCR)-based methods. *Diagnostic Microbiology and Infectious Disease*.

[29] Van De Peer Y, De Wachter R (1994). Treecon for windows: a software package for the construction and drawing of evolutionary trees for the microsoft windows environment. *Bioinformatics*.

[30] Aragão FJL, Brasileiro ACM (2002). Positive, negative and marker-free strategies for transgenic plant selection. *Brasilian Journal of Plant Physiology*.

[31] Strandén A, Frei R, Widmer AF (2003). Molecular typing of methicillin-resistant *Staphylococcus aureus*: can PCR replace pulsed-field gel electrophoresis?. *Journal of Clinical Microbiology*.

[32] Walker ES, Vasquez JE, Dula R, Bullock H, Sarubbi FA (2003). Mupirocin-resistant, methicillin-resistant *Staphylococcus aureus*: does mupirocin remain effective?. *Infection Control and Hospital Epidemiology*.

[33] Simor AE, Stuart TL, Louie L (2007). Mupirocin-resistant, methicillin-resistant *Staphylococcus aureus* strains in Canadian Hospitals. *Antimicrobial Agents and Chemotherapy*.

[34] Vasquez JE, Walker ES, Franzus BW, Overbay BK, Reagan DR, Sarubbi FA (2000). The epidemiology of mupirocin resistance among methicillin-resistant *Staphylococcus aureus* at a Veterans’ Affairs hospital. *Infection Control and Hospital Epidemiology*.

[35] Jones JC, Rogers TJ, Brookmeyer P (2007). Mupirocin resistance in patients colonized with methicillin-resistant *Staphylococcus aureus* in a surgical intensive care unit. *Clinical Infectious Diseases*.

[36] Cookson BD (1998). The emergence of mupirocin resistance: a challenge to infection control and antibiotic prescribing practice. *Journal of Antimicrobial Chemotherapy*.

[37] Boers SA, Van Ess I, Euser SM, Jansen R, Tempelman FRH, Diederen BMW (2011). An outbreak of a Multiresistant Methicillin-Susceptible *Staphylococcus aureus* (MR-MSSA) strain in a Burn Centre: the importance of routine molecular typing. *Burns*.

[38] Nannini E, Murray BE, Arias CA (2010). Resistance or decreased susceptibility to glycopeptides, daptomycin, and linezolid in methicillin-resistant *Staphylococcus aureus*. *Current Opinion in Pharmacology*.

[39] Sola C, Lamberghini RO, Ciarlantini M (2011). Heterogeneous vancomycin-intermediate susceptibility in a community-associated methicillin-resistant *Staphylococcus aureus* epidemic clone, in a case of Infective Endocarditis in Argentina. *Annals of Clinical Microbiology and Antimicrobials*.

